# High-Throughput Genotype, Morphology, and Quality Traits Evaluation for the Assessment of Genetic Diversity of Wheat Landraces from Sicily

**DOI:** 10.3390/plants8050116

**Published:** 2019-04-30

**Authors:** Maria Carola Fiore, Francesco Mercati, Alfio Spina, Sebastiano Blangiforti, Gianfranco Venora, Matteo Dell’Acqua, Antonio Lupini, Giovanni Preiti, Michele Monti, Mario Enrico Pè, Francesco Sunseri

**Affiliations:** 1 CREA Research Centre for Plant Protection and Certification, 90011 Bagheria (PA), Italy; mariacarola.fiore@crea.gov.it; 2 National Research Council of Italy, Institute of Biosciences and Bioresources (CNR-IBBR), 90129 Palermo, Italy; 3 CREA Research Centre for Cereal and Industrial Crops, Acireale (CT) 95024, Italy; alfio.spina@crea.gov.it; 4 Stazione Consorziale Sperimentale di Granicoltura per la Sicilia, 95041 Caltagirone (CT), Italy; blangiforti@granicoltura.it (S.B.); venora@granicoltura.it (G.V.); 5 Institute of Life Sciences, Scuola Superiore Sant’Anna, 56127 Pisa, Italy; matteo.dellacqua@santannapisa.it (M.D.); marioenrico.pe@santannapisa.it (M.E.P.); 6 Dipartimento Agraria, Università Mediterranea di Reggio Calabria, 89021 Reggio Calabria, Italy; antonio.lupini@unirc.it (A.L.); preiti@unirc.it (G.P.); montim@unirc.it (M.M.)

**Keywords:** wheat landraces, genetic diversity, single nucleotide polymorphism, morphological and quality traits

## Abstract

During the XX Century, the widespread use of modern wheat cultivars drastically reduced the cultivation of ancient landraces, which nowadays are confined to niche cultivation areas. Several durum wheat landraces adapted to the extreme environments of the Mediterranean region, are still being cultivated in Sicily, Italy. Detailed knowledge of the genetic diversity of this germplasm could lay the basis for their efficient management in breeding programs, for a wide-range range of traits. The aim of the present study was to characterize a collection of durum wheat landraces from Sicily, using single nucleotide polymorphisms (SNP) markers, together with agro-morphological, phenological and quality-related traits. Two modern cv. Simeto, Claudio, and the hexaploid landrace, Cuccitta, were used as outgroups. Cluster analysis and Principal Coordinates Analysis (PCoA) allowed us to identify four main clusters across the analyzed germplasm, among which a cluster included only historical and modern varieties. Likewise, structure analysis was able to distinguish the ancient varieties from the others, grouping the entries in seven cryptic genetic clusters. Furthermore, a Principal Component Analysis (PCA) was able to separate the modern testers from the ancient germplasm. This approach was useful to classify and evaluate Sicilian ancient wheat germplasm, supporting their safeguard and providing a genetic fingerprint that is necessary for avoiding commercial frauds to sustaining the economic profits of farmers resorting to landraces cultivation.

## 1. Introduction

Wheat (*Triticum* spp.) is the largest primary commodity and one of the oldest and most important cereal crops, being grown on more land area than any other crop, worldwide. Durum wheat (*Triticum turgidum* L. subsp. *durum* Desf.) is the second most important *Triticum* species, next to common wheat (*Triticum aestivum* L.), and is mainly used to produce semolina and dried pasta. The European Union (EU) holds the largest share in world durum wheat harvested production (8956.58 thousand tons), Italy being the main EU producer with 4303.10 thousand tons (EUROSTAT, 2018; https://ec.europa.eu/eurostat/web/agriculture/data/database). Durum wheat is a crop of primary importance for the Mediterranean region and is mainly cultivated under rainfed conditions, resulting in major yield variations, due to irregular rainfalls. Outside of the Mediterranean region, durum wheat is cultivated in Northern America (Canada, USA.) and Central America (Mexico), as well as in smaller growing areas of Russia, Australia, Argentina, and Ethiopia.

Durum wheat is a member of the *Gramineae*, included in the *Triticeae* tribe, and it belongs to the *Triticum* genus. Based on cytological and molecular analyses, *Triticum turgidum* is believed to have originated from a hybridization between *Triticum urartu* (AA genome, n = 7) and an unknown diploid species (BB genome, n = 7) closely related to *Aegilops speltoides* [1]. Thus, durum wheat is a self-pollinated allotetraploid cereal (harboring two genomes with genomic formula: AABB) with 28 chromosomes (2n = 4x = 28). All *Triticum* species are native to the Fertile Crescent of the Near East [1]. In particular, the Jordan River valley and Levant Corridor, quite a narrow strip between the Mediterranean Sea to the northwest and deserts to the southeast, localize the origin of domesticated tetraploid wheats, including the durum wheat [1]. Wheat cultivation had spread westwards to Europe (Italy, France, and Spain) and North Africa, during the Neolithic period through Anatolia and Greece. Although wheat was introduced in Sicily during the ancient Greek colonization, it was only during the Roman domination in the third century BC that Sicily become an important *Granary of Rome*.

At the beginning of the last century, durum wheat breeding was pioneered in Italy when durum and common wheat started to differ more clearly [2]. Many tetraploid wheat landraces were already present in Italy, when different exotic landraces were introduced and included into the breeding programs [3,4]. In the first 20 years of the XX century, the Nazareno Strampelli research activity led to firstly development of cultivars, based on the selection of available diversity. The main results were achieved through the development of pure lines for breeding programs starting from the local races of Southern Italy. The continuation of Strampelli’s project by many Italian breeders led to the release of several new varieties in the period of 50s to 60s, through the exploitation of the large genetic variability of the available genetic stocks [5]. These cultivars already harbored the GA-insensitive dwarfing genes introgressed from North African wheat germplasm. The intensive activities of wheat breeding in Italy continued during the second half century, at the Agricultural Experimental Stations and the Academic Institutions, mainly to improve crop yield and quality with a particular emphasis on traits related to plant adaptation.

The spread of modern wheat cultivars drastically reduced the cultivation of the “old landraces”, confined to the niche areas of cultivation, with their maintenance being entrusted to public research institutions and custodian farmers. Nowadays, these landraces represent a collection of important genetic resources with traits of biological and economic significance, as they have been subjected to several cycles of artificial and natural selections, as reported by De Cillis [6]. The preservation of “historical cultivars” or landraces could be important to avoid genetic erosion, fostering their utilization in the new breeding programs [7]. Indeed, a higher tolerance of ancient landraces against biotic and abiotic stresses, compared to the modern varieties has been demonstrated [8,9]. Nowadays attention is given to the cereals nutritional values and health effects and the terms “ancient grains”, “historical varieties”, “modern varieties” have become common in the scientific community and among consumers, covering a broad subject area, including the identification of varieties. Growing consumer interest in healthy food production has triggered interest in the phytochemical content of ancient and modern durum wheat varieties [10], and this trend led to the rediscovery and re-utilization of durum wheat landraces, including those in Sicily [11].

Knowledge on genetic diversity of durum wheat landraces should be the starting point for their utilization and conservation management. Wheat genetic diversity has been mostly evaluated using morphological and phenological descriptors, established by the International Plant Genetic Resources Institute (IPGRI) [12] and the International Union for the Protection of New Varieties of Plants (UPOV) [13], in addition to the assessment of variations in seed storage proteins. Nowadays, molecular markers represent a powerful tool to analyze genetic diversity and could also aid to maintain germplasm, with a minimum repetition of genotypes, for conservation. Among molecular markers, single nucleotide polymorphisms (SNP) [14,15,16] are point mutations that result in single base-pair divergence, among DNA sequences present in both genome coding and noncoding regions. The advent of high-throughput Next Generation Sequencing (NGS) technologies enabled the large-scale identification of SNPs in plant species, and the development of efficient genotyping platforms, for association studies [17,18] and genetic characterization, also in wheat [19,20,21,22,23,24,25,26], where the tetraploid condition make the utilization of microsatellites difficult.

Among Mediterranean regions, Sicily is one of the richest in wheat landraces diversity. In Sicily, several durum wheat landraces were recently re-discovered and re-employed because of their adaptability to extreme Mediterranean environments, due to their genetic structure, typical of a self-pollinated population (mixture of different pure lines). The Experimental Sicilian Station for Durum Wheat (ESS) at Caltagirone (province of Catania, Italy), is the wheat research station responsible for the conservation of local varieties, breeding and research for wheat growers, and processors in Sicily. The ESS bred and preserved over 200 accessions along the years, including 48 local varieties of *Triticum durum*, *T. aestivum*, and *T. turanicum*, previously collected and described thanks to the effort of De Cillis [6]. The germplasm collection at Caltagirone is being maintained through pure seed multiplication, for almost 15 years now. Some of these landraces are still being cultivated in Sicily, while others represent unique genetic resources to be exploited in future breeding programs. In the panel, varieties that largely spread in the last century in Sicilian wheat growing areas were included, among which “Cappelli” has been registered in the Italian National Register of varieties in 1915 and is still widely cultivated, and “Trinakria”, has been registered in 1970 but has currently phased out.

The aim of this study was to evaluate the genetic diversity in a wheat collection from the ESS, using the SNP markers, and agro-morphological, phenological, and quality-related traits. A detailed evaluation and classification of the Sicilian durum landraces will allow their proper preservation, promote their sustainable use, and eventually provide a reproducible genetic fingerprint that can be used for avoiding commercial frauds and sustaining the economic profits of the farmers.

## 2. Results and Discussion

### 2.1. Morphological and Grain Quality Traits

**Figure 1:**
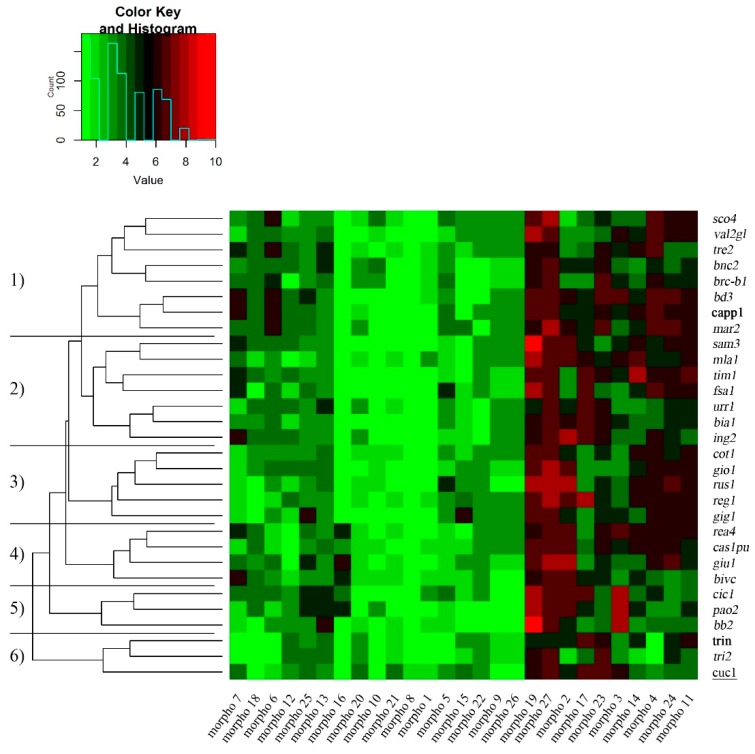
Heatmap of the morphological traits measured on Sicilian wheat germplasm (27 landraces; in italic), 2 historical varieties (Cappelli and Trinakria; in bold) and 1 bread wheat landrace (Cuccitta; underlined). Green and red colors represent reduced and augmented representation levels, respectively. Hierarchical clustering of samples (cluster 1–6) and traits are also shown. All the traits, coded as morpho 1 to morpho 27, are described in Table S1.

Durum wheat collection effectively preserved and documented at the ESS were characterized by their different morphological, phenological and grain quality traits. A heatmap was generated for a simplified representation of the morphological trait distribution (Figure 1; Table S1).

Overall, these traits allowed us to group the germplasm in five main clusters, with 50% of landraces/cultivars belonging to cluster 1 and 2, while 5, 4, 3, and 3 genotypes belonged clusters 3, 4, 5, and 6, respectively. Interestingly, ten traits showing high variability among the samples, as determined by their clusters—six traits (morpho 2, morpho 11, morpho 19, morpho 23, morpho 24, and morpho 27; Table S1) were mainly due to the genotypic variation; while three traits were highly influenced by environment (morpho 3, morpho 4, and morpho 17; Table S1). In addition, flag leaves (morpho 2) played a very important role in grain filling and yield, together with ear emergence (morpho 3), ear cross shape (morpho 23), and density (morpho 24).

The reduction of plant height, together with a higher grain yield and early maturation, were the main breeder aims in the last century. Our historical Sicilian wheat collection, investigated here, showed mostly a growth erect or semi erect (data not shown), and the plant height ranged from 95 and 135 cm. Nineteen percent of the landraces showed a plant height below 120 cm (Table S1). Based on the data collected, the time of ear emergence, counted as days from sowing to flowering, ranged from early (120 days) to medium (125 days) in more than 70% of the collection (Table S1). Among landraces, it was possible to recognize genotypes belonging to the ‘*syriacum typicum*’, a group utilized to select and release shorter and earlier-flowering new cultivars in Italy, during 1920–1950 [27]. These results supported previous reports [28], confirming the key role of these genetic resources for a potential re-introduction of the historical durum wheat landraces in cultivation.

Mains comparison and post-hoc test of grain and wholemeal flour quality-related (commercial) traits of wheat genotypes are reported in Table 1 (one-way ANOVA is reported in Table S2).

In durum wheat that have not undergone weathering, test weight (TW) is an excellent predictor of the semolina milling potential. Indeed, this parameter indicated the specific seed weight per unit volume, depending on the filling degree of the seed, was used to indicate the likely milling performance of wheat, and was considered a more reliable predictor of the flour/semolina yield [29,30]. Here, there were no significant differences between the highest value recorded for cv. Claudio and those measured in the two landraces (val2gl and tri2). The same was applicable for the other five landraces (urr1, tre2, trin, sam3, and bivc), when compared to the value recorded for cv. Simeto. The thousand-kernel-weight (TKW) ranged from 33.5 to 59.2 g recorded in “tim” and “val2gl”, respectively. Interestingly, thirteen out of the twenty-seven landraces recorded significant TKW higher values, as compared to cv. Claudio, furthermore, seven landraces also showed a higher TKW value than the modern cv. Simeto. These performances might be due to earlier flowering of modern wheat cultivars, as previously reported [31]. These results indicate that the durum wheat landraces from Sicily might possess a higher yield potential than the modern cultivars, when cultivated under drought stress conditions.

Non-vitreous kernels (starchy), characterized by an opaque area, have a significant impact on the characteristics of kernels during milling, producing a detrimental effect to the end-use quality of durum wheat, and decreasing the semolina extract [32]. The incidence of starchy kernels, recorded on the Sicilian wheat collection, ranged from 0 (sco4) to 96% (tri2), with the landraces showing a higher percentage, compared to the modern cultivars, as reported in Gallo et al. [33].

Wholemeal flour quality traits, protein and gluten contents are the most important features useful for characterizing durum wheat landraces/cultivars. Indeed, it is well-documented that the protein content and the endosperm storage protein composition have a decisive impact on the wheat processing quality [34,35]. High protein level in semolina will usually yield a product with uniform particle size, with a minimum number of starchy particles, although it has been shown that other traits, like such specific γ-gliadins and gluten viscoelasticity, together with vitreousness and yellow semolina color, play a pivotal role in quality, processed wheat products [36,37].

Frequently, modern durum wheat cultivars show lower grain protein content, compared to the older ones [38,39,40], as the wheat breeding programs are mainly focused on increasing the grain yield [41]. Our result seems to confirm these previous reports. Indeed, a significantly higher protein content (>16.0%) was observed in the six Sicilian landraces (bd3, sco4, reg1, cic1, gig1, and ing2) compared to the modern cultivars (<15%), whereas the lowest value (8.5%) was found in tri2, due to the high percentage of starchy kernels (Table 1).

The values of wet and dry gluten content, important for the pasta industry, showed a comparable trend (r^2^ = 0.97) with a high protein content and a wide variability, as already reported [42,43]. The historical cv. Capp1, recently registered again in the Italian National Register of Varieties (INRV) for its reintroduction in the wheat cultivation areas of Italy, showed a very high value of wet gluten content (38.1%). With regard to the gluten index, often correlated with gluten quality, most of the ancient wheat landraces showed low values, ranging from 18.8 (fsa1) to 59.5 (cot1) (Table 1; Figure S1). By contrast, “mla1” landrace showed a significant higher value (91.12), compared to the testers (86.3 and 83.1 in Claudio and Simeto, respectively). These data confirmed the presence of a weaker gluten in Sicilian wheat landraces, as previously reported [44,45,46,47], compared to the modern cultivars [48,49,50].

Durum wheat grain color might affect the quality of the end products, depending on genetic background of a genotype, as well as the environment and the technological processes. The anthocyanins are a class of pigments, which characterize the durum wheat aleurone or pericarp. The high-level of these pigments observed in many Sicilian landraces represents an important trait for breeding programs aimed at improving the nutritional value of grain and its end products [51]. The yellow index (b*) measured in semolina was significantly higher in the cv. Simeto (20.06), compared to those measured in the Sicilian landraces, demonstrating the effect of selection on this important nutritional trait. The historical cv. Cappelli (capp1), together with two landraces, “fsa1” and “rea4” were characterized by a high value of yellow index (b*), according to Digesù et al. [52]. As expected, the bread wheat landrace (cuc1) showed the significant lowest yellow index (8.74).

**Figure 2:**
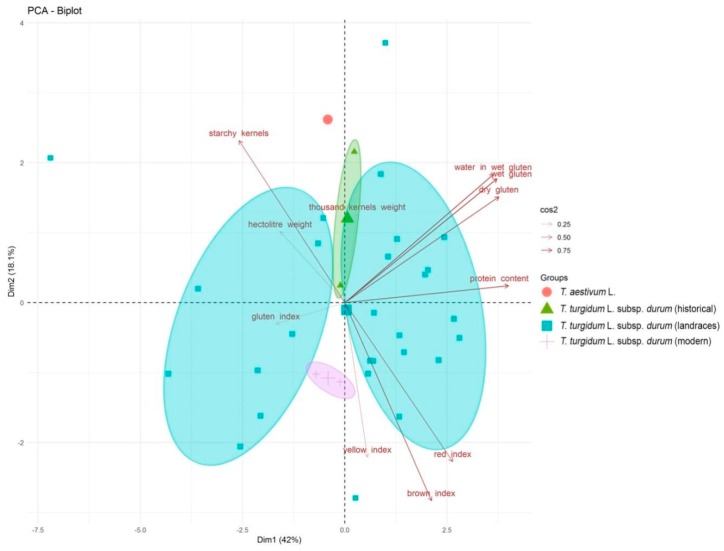
Principal Component Analysis (PCA) of the quality-related traits. Based on their origin, samples were organized in four main groups: (1) *T. aestivum* L., (2) *T. turgidum* L. subsp. *durum* historical varieties (Cappelli and Trinakria), (3) *T. turgidum* L. subsp. *durum* ancient landraces (27), and (4) *T. turgidum* L. subsp. *durum* modern varieties (Claudio and Simeto). Traits associated with the samples discrimination are indicated in the plot, underlining their significance values (0.25 < cos2 < 0.75). The medians for each group were also visualized.

The Principal Component Analysis (PCA) performed on all the quality-related grain traits showed wide differences among landraces (Figure 2). The first two components explained 60.1% of the total variance, the second ones were able to discriminate cuc1 (*T. aestivum* L.) from the landraces belonging to *T. turgidum* L. *ssp*. *durum*, and the last components were able to discriminate between historical and modern cultivars (Figure 2). Protein content and the parameters related to gluten showed a strong influence on both components (cos2 = 0.75), separating 50% of landraces (16) from the others. Finally, *T. aestivum* L. was characterized by a higher number of starchy kernels (cos^2^ = 0.75), strongly related to the first component (Figure 2). These evidences were confirmed by Pearson correlations, showing a positive correlation (*p* < 0.05) among water binding in wet gluten, and dry and wet gluten, with ae higher positive correlation coefficients (0.96) for the last two components and a negative correlation (*p* < 0.05) between starchy kernels and protein content (−0.64) (Figure S2). Likewise, the red, brown, and yellow index were positive correlated (*p* < 0.05).

### 2.2. SNP Analysis and Genetic Characterization

Genetic diversity can be evaluated by biochemical and morphological markers or by the use of pedigree. Unfortunately, these approaches can be influenced by environment or can be erroneous and incomplete, causing frequent misclassifications among the genotypes. By contrast, molecular markers allow the assessment of relatedness at the DNA level, making them necessary for identification of genetic variation among and within landraces/populations, due to the influence of the environment on many traits under polygenic control. Different molecular markers can be used for comparative genomic, phylogenetic relationships, and diversity studies [53,54]. More recently, next generation sequencing (NGS) led to set up different SNP panels that were successfully used for genetic diversity analyses, also in wheat [21,55]. The high-throughput wheat 90k SNP array was used to investigate the genetic relationships across the Sicilian ancient germplasm, using two modern cultivars (Claudio e Simeto) as references, and Cuccitta, a bread wheat landrace, as the outgroup. After the SNP-dataset filtering, 5,594 loci (7%) did not amplify among all genotypes and 41,926 (51%) were monomorphic (Table 2). The final dataset resulted in 18,170 loci, after removing the SNPs with a number of NC (not-call) higher than 20%, among which 13,528 loci (74%) were polymorphic with an overall Minor Allele Frequency (MAF) value of 0.232 (Table 2).

**Figure 3:**
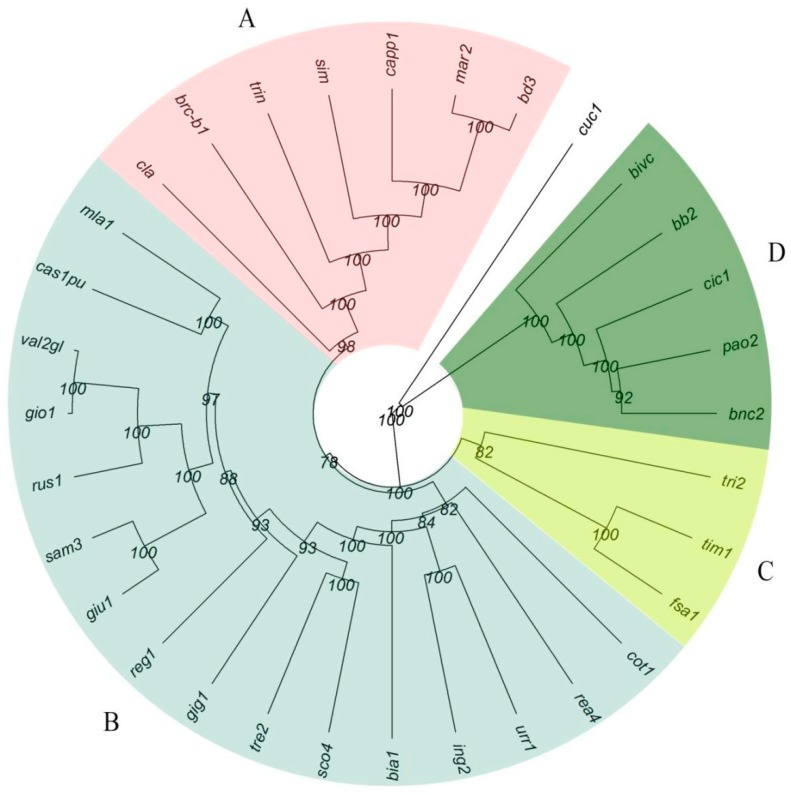
Genetic relationships among ancient landraces and the two historical varieties Cappelli (capp1) and Trinakria (trin), belonging to the Sicilian wheat germplasm collection, obtained by using 18,170 SNPs. Simeto (Sim) and Claudio (Cla) were utilized as testers, while the hexaploid Cuccitta was added as an outgroup. Dendrogram generated using the UPGMA method and Nei’s distance.

To investigate the genetic relationships among cultivars, based on the SNP-data, phylogenetic analysis and PCoA were carried out. Cluster analysis based on Nei (1978) genetic coefficient and the UPGMA algorithm, generated a dendrogram underlining four main clusters across the durum wheat germplasm (Figure 3). Two modern and the historical cultivars (Claudio and Simeto; Trinakria and Cappelli, respectively) grouped in Cluster A, together with three landraces (brc-b1, mar2, and bd3), while the bread wheat landrace Cuccitta was the outgroup, as expected (Figure 3). Cluster B included 16 out of 27 durum wheat landraces (60%), while cluster C and D grouped only three and five landraces, respectively. Many bootstrap values (80%) ranged from 99 to 100% in the most important nodes, avoiding any misclassifications (Figure 3). Cluster A was closer to Cluster B (NEI = 0.1552), while Cluster D appeared more different from the other three (Table S3). As expected, the outgroup Cuccitta (cuc1) showed the highest values of genetic distance from the other groups (Table S3).

PCoA was also carried out to properly describe the clusters reported above, as expected, the first two PCs showed four main clusters, grouping the different genotypes in agreement with cluster analysis (Figure S3). Genetic distances among genotypes were confirmed, with five landraces belong to cluster D (Figure 3) showing the highest genetic variability, and the landraces grouped in cluster B and C showing a common genetic background. As expected, cuc1 landrace was separated from the others. Unlike cluster analysis, brc-b1 appeared similar to cuc1, between the samples belonging to cluster A and D, without a clear assignment (Figure S3).

**Figure 4:**
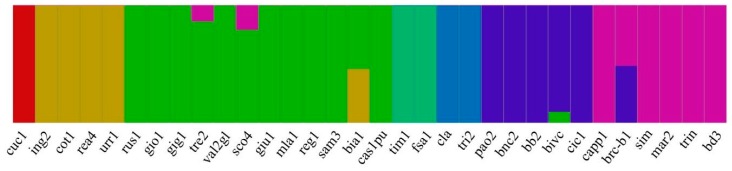
Admixture proportions of the wheat germplasm collection estimated by the FastStructure (*K* = 7). Each vertical bar represents a sample. The color proportion for each bar represents the posterior probability of assignment of each individual to one of the seven groups of genetic similarity. The range of assignment probability varied from 0 to 100%.

The differences among cultivars by fast STRUCTURE analysis were further confirmed. Indeed, although the groups highlighted by the two approaches were different, this analysis confirmed cluster and PCoA results, and was able to separate the ancient cultivars from the others. The optimum number of genetic clusters (*K*) within the collection was determined as *K* = 7 (Figure 4). The Cuc1 hexaploid landrace belonged to a private group (red pool; outgroup in the cluster analysis) and the other two pools, green and gold, overlapped the clusters B defined in the UPGMA analysis (clustering), with ing2, cot1, rea4, and urr1 varieties being grouped in private branches. Likewise, the blue pool of the structure analysis overlapped with cluster D. By contrast, cluster A in the UPGMA, harbored the purple pool, except the cultivar Claudio which shared a different genetic pool (light blue) with tri2, in the structure analysis (Figure 4). However, cla and tri2 samples were outside their main clusters, and genetically closer to each other, in comparison to the samples belonging to groups B and D. Thirty out of the 32 genotypes (94%) classified into one of the seven pools, using an 80% cut-off ancestry. Thus, many varieties showed 100% membership to their group (*K*), except bivc, sco4, and tre2. Among landraces, only brc-b1 and bia1 showed a high admixture profile between the gold and green, the purple and blue pools, respectively (Figure 4).

To prioritize germplasm within the collection, a genetic distinctness and a genetic redundancy analyses were run using R/AveDissR. In the distinctness analysis, the average dissimilarity (AD) values ranged from 0.31 to more than 0.57, the most distinct individual being cuc1 (Table S4). Selecting the 15 samples with higher AD in the sixth step of the iteration resulted in a percentage of variation (PVa) of 0.13 among the populations, as compared to the initial (0.10). Any further selection with the chosen step of 0.1 would substantially reduce the PVa (Table S5), hindering the representativeness of the most distinct genotypes. Genetic redundancy was assayed to identify the least unique samples. PVa dropped from 0.10 to 0.08, when removing the three samples with the lower AD values (Table S6), indicating a slight reduction from the overall genetic differentiation of the collection. The outcome of R/AveDissR analysis might be used to guide the selection of the most distinct genotype, which, in combination with passport information and further phenotypic characterization, might contribute to breeding programs willing to employ these genetic resources. By contrast, the identification of samples characterized by lower AD values would allow to further characterize the redundant genotypes putatively derived from the shared ancestry or even from the duplications in the seed bank.

## 3. Conclusions

Genetic variation in wheat landraces was often analyzed by using morphological and biochemical markers [56], but their polygenic control suggested the utilization of molecular markers, for detecting genetic variation and characterizing germplasm collections [57].

Here, we investigated a collection of durum wheat landraces by using morphological and quality-related traits, together with a large SNP panel [21]. The germplasm collection is effectively conserved and available at the ESS of Caltagirone (Catania, Italy), a large treasure of wheat genetic biodiversity that includes the landraces from the Nazareno Strampelli research activity, which has been described well by De Cillis [6]. In the last forty years, wheat landraces have been replaced by modern cultivars, although they can exhibit wide adaptations to climatic extremes and tolerance to abiotic and biotic stresses. Plant height, general late maturity, and low harvest index have limited their cultivation to a few marginal areas. Thus, the re-use of landraces in cultivation or in breeding programs could improve the resilience of wheat, due to the morphological traits that are useful for tackling the limited resources available in the marginal areas and in the organic agricultural systems. Our study focused on the assessment of the genetic potential of durum wheat landraces from Sicily as an important source of variation, and on detecting unexplored alleles.

SNPs appeared powerful for evaluating genetic diversity and classifying different durum wheat landraces, due to their reproducibility and the development of relatively high-throughput platforms. Cluster analysis based on the Nei (1978) genetic coefficient and UPGMA algorithm generated a dendrogram underlining the genetic relationships among landraces/cultivars, distinguishing four main clusters across durum wheat germplasm. PCoA confirmed the clusters with the first two PCs highlighting four main groups of genotypes. SNP were also able to discriminate among landraces and within populations by isolating a panel of SNP able to distinct haplotypes. This ability might be useful to define gene-banks conservation strategies by avoiding genetic erosion through selection. The SNP panel will allow us to organize an efficient system for genetic traceability of wheat end products, eluding commercial frauds and sustaining economic profits for the farmers.

In light of our results, the interesting panel of investigated genotypes could represent a platform for parental selection to develop high-yield durum wheat lines in breeding programs and for association mapping studies.

## 4. Materials and Methods

### 4.1. Plant Material and Experimental Conditions

Twenty-seven durum and one bread wheat Sicilian landrace from the collection of the “Experimental Sicilian Station for Durum Wheat” (ESS) of Caltagirone (Catania), two historical cultivars (Cappelli and Trinakria) parents of many Italian breeding programs, and two modern cultivars (Claudio and Simeto), widespread in Sicilian cereal growing areas were included in this study (Table 3).

Field trials were carried out in 2013 and 2014 in Sicily (Caltagirone, Catania province, 37° 05′ 58′’ N., 14° 29′ 56′’ E., 280 m a.s.l.) in a medium-sandy soil, to assess the main bio-productive traits and quality of the grain and whole grain flours. Genotypes were laid out in the field in 10 m^2^ plots, according to a randomized blocks experimental design with three replicates, adopting the typical wheat agronomic management.

### 4.2. Morphological Traits Characterization

In accordance with the Community Plant Variety Office descriptors [13], twenty-seven morphological and physiological traits were evaluated for each landrace and reference cultivars. Data were collected on ten plants, during vegetative growth, using the Zadoks scale system from stage 10 (first leaf through coleoptile) to stage 60 (full heading but not flowering) and after harvest, on a random sample of fifteen representative spikes (five in each replicate), randomly sampled from each landrace/cultivar.

### 4.3. Grain and Wholemeal Flour Quality-related Traits

Samples of durum wheat grains per each plot were analyzed for the main quality traits. Protein content (% dry matter) was determined by means of Infratec 1241 Grain Analyzer (Foss Tecator, Höganas, Sweden) by near infrared transmittance (NIT), using a calibration based on the Kjeldahl nitrogen method (UNI EN ISO 20483). Calibration was validated in accordance with UNI EN ISO 12099 [58], using different sets of test samples of durum wheat grain with a linear correlation coefficient of r = 0.99.

Further, representative samples of grains per plot were used to measure the thousand kernels weight (TKW), test weight (TW), and the starchy kernels. TKW was obtained by weighting 8 samples of 100 seeds each and the average weight was compared to 1,000 seeds.TW was determined with a Test Weight Module (TWM) installed under the Infratec 1241 Grain Analyzer (Foss Tecator, Höganas, Sweden). The visual estimation of the starchy kernel percentage was carried out on a representative seed sample (30 g). Kernels were sorted visually into wholly vitreous grains and not (at least two spot) and this last was expressed as a percentage of the total seeds. Worldwide recognized procedures (ISO and ICC standard method No. 129) defined fully vitreous kernels as “those that do not disclose the least trace of farinaceous endosperm” [59].

Grain obtained from each treatment was milled to obtain wholemeal flour by an experimental mill, Cyclotec type 120 (Falling Number, Huddinge, Sweden), with a sieve of 0.5 mm. Physical and chemical gluten features were analyzed by using a Glutomatic 2200, a Centrifuge 2015, and a Glutork 2020 (Perten Instruments AB, Huddinge, Sweden). Wet and dry gluten content and quality (gluten index) were calculated according to the ICC Standard No. 158 [60]. Water binding capacity (WBC) was calculated as the difference between the weight of the total wet and dry gluten, which gave the water bound in the wet gluten, according to AACCI 38-12.02 [60]. Colorimetric measurements on wholemeal flours were performed following the method described by Sgrulletta et al. [61], which were expressed using the standard CIE 1976 L*a*b* system [62]. Accordingly, the measured indices were: L* (lightness in the range between black = 0 and white = 100), a* (the difference between red and green tones), and b* (direct measurement of the yellow color). These indices were obtained by means of a CR200 Minolta Colorimeter Chroma (Minolta, Osaka, Japan), using illuminant D_65_ and a* and b* corresponded directly to the red and yellow indices, respectively, while the brown index was obtained as 100-L*. Protein, wet and dry gluten content, WBC, and starchy kernels were analyzed using the arc sine transformation [63], prior to the analysis of variance.

### 4.4. Statistical Analysis for Morphological and Quality-Related Traits

Samples of wheat grains were analyzed for eleven main quality and commercial traits—protein content, thousand kernels weight, test weight, starchy kernels, wet and dry gluten content, water binding in wet gluten, gluten index, brown index, yellow index, and red index. A Hierarchical analysis of variance (ANOVA) was calculated and a post hoc Tukey’ test was adopted to compare the means of 32 durum and bread wheat landraces/cultivars using XLSTAT version 2018.3. Percentage data were normalized expressing them as arc sin root square. Percentage data of starchy kernels, protein content, wet and dry gluten contents were normalized, and expressed as arc sin root square.

Principal Component Analysis (PCA) was performed using the R package FactoMiner [64]. Finally, the Pearson correlation coefficient (*p* < 0.05) was also calculated among the quality-related traits using the Hmisc R/package (https://cran.r-project.org/web/packages/Hmisc/index.html) and a scatter plot with the correlation coefficients and their significance was developed with the Performance Analytic R/package (https://cran.r-project.org/web/packages/PerformanceAnalytics/index.html).

### 4.5. DNA Extraction and SNP Genotyping

Genomic DNA was extracted from the fresh leaves/young seedlings of each landrace and cultivar. The GenElute Plant Genomic DNA Miniprep Kit (Sigma-Aldrich, St. Louis, MO, USA) was used and the DNA quantity and quality were checked by electrophoresis (1% agarose gel) and NanoDrop^®^ ND-1000 (Thermo Scientific, Walthman, MA, USA), respectively. DNAs were delivered to TraitGenetics GmbH (Gatersleben, Salzlandkreis, Germany) for genotyping. Two hundred nanograms of genomic DNA were used as a template for each reaction, following the manufacturer’s instructions (Illumina Inc., San Diego, CA, USA). SNP data matrix was generated from the custom Illumina by wheat 90k SNP array (Illumina Inc., San Diego, CA, USA), which assays 81,587 SNPs [21].

### 4.6. 90K Chip Array SNP-data Analysis

Raw data were visualized and analyzed by using GenomeStudio V2011.1 software (Illumina), with the polyploid clustering version 1.0.0. The dataset was filtered and standardized as previously reported [65]. SNPs with MAF > 0.05 and missing rate < 0.20 were used for all subsequent analyses. The final dataset used for analysis was reported in Table S7. MAF, polymorphic loci and genetic distance (NEI’s 1972) were evaluated by PLINK [66], R/snpStats [67] and R/HierFstat packages [68], respectively. To investigate the genetic relationships among landraces/cultivars, cluster analysis and Principal Coordinates Analysis (PCoA) were carried out by using R/adegenet 1.3 [69]. To highlight the number of putative genetic pools (*K*) available in Sicilian wheat germplasm, FastStructure in the admixture model was used [70]. The analysis was performed as described in Mercati et al. [71], using the input files (.bed, .bim, .fam) generated by PLINK, following the default parameters (http://rajanil.github.io/fastStructure/). To assess the genetic distinctness and redundancy among samples, R/AveDissR [72] was used.

## Figures and Tables

**Table 1:** Means comparison and post hoc test (Tukey’s) for grain and wholemeal flour quality-related parameters of Sicilian wheat germplasm collection.

ID	Test Weight (kg/hL)	Thousand Kernels Weight (g)	Starchy Kernels (%)	Protein Content (% d.m.)	Wet Gluten Content (%)	Dry Gluten Content (%)	Water Binding in Wet Gluten	Gluten Index (0–100)	Brown Index (100^−^L*)	Red Index (a*)	Yellow Index (b*)
**bia1**	79.2 ± 0.14^e^	41.9 ± 0.14^q^	2.5 ± 0.71^ln^	15.5 ± 0.07^a^	36.00 ± 0.57^eg^	11.10 ± 0.2^eg^	24.90 ± 0.28^e^	48.60 ± 0.81^J^	17.75 ± 0.0^b^	1.44 ± 0.03^bc^	14.80 ± 0.11^lm^
**bd3**	77.6 ± 0.14^gh^	39.2 ± 0.14^r^	5.5 ± 0.71^il^	17.1 ± 0.00^a^	42.45 ± 0.64 ^b^	14.70 ± 0.28^a^	27.75 ± 0.35^c^	56.65 ± 0.65^eg^	14.84 ± 0.12^mn^	0.59 ± 0.09^ef^	15.57 ± 0.01^ij^
**bivc**	80.2 ± 0.28^d^	54.9 ± 0.07^d^	43.5 ± 2.12^cd^	14.1 ± 0.07^e^	32.25 ± 0.92 ^il^	9.65 ± 0.21^jk^	22.60 ± 0.71^gh^	36.10 ± 1.82^mn^	15.64 ± 0.18^ij^	0.60 ± 0.01^ef^	14.15 ± 0.20^no^
**bb2**	71.8 ± 0.14^q^	45.3 ± 0.14^no^	15.5 ± 2.12^f^	14.9 ± 0.07^cd^	31.45 ± 0.92^jl^	10.10 ± 0.28^hj^	21.35 ± 0.64^gi^	54.51 ± 1.33^fh^	16.63 ± 0.24d^e^	0.70 ± 0.08^de^	14.06 ± 0.0^o^
**bnc2**	73.9 ± 0.07^no^	43.2 ± 0.14^p^	9.5 ± 2.12^gi^	14.1 ± 0.00^e^	34.40 ± 1.13^gi^	9.95 ± 0.35^hj^	24.45 ± 1.48^ef^	37.18 ± 2.07^mn^	15.72 ± 0.01^hi^	1.44 ± 0.06^bc^	13.36 ± 0.12^p^
**brc-b1**	75.6 ± 0.14^l^	49.2 ± 0.21^ij^	28.5 ± 2.12^e^	14.1 ± 0.07^e^	42.25 ± 0.50 ^b^	11.80 ± 0.14^ce^	30.45 ± 0.35^b^	48.40 ± 0.60 ^j^	14.50 ± 0.06^n^	1.01 ± 0.06^d^	15.50 ± 0.09^ik^
**capp1**	81.4 ± 0.07^b^	49.6 ± 0.58^i^	9.0 ± 1.41^gi^	15.1 ± 0.00^ab^	38.10 ± 0.85 ^ce^	12.00 ± 0.14^cde^	26.10 ± 0.71^cde^	19.67 ± 1.79^q^	13.87 ± 0.35^o^	0.89 ± 0.0^d^	18.24 ± 0.69^c^
**cas1pu**	76.8 ± 0.07^ik^	49.7 ± 0.21^i^	1.0 ± 0.00^no^	14.8 ± 0.07^d^	32.00 ± 1.27 ^il^	10.00 ± 0.1^hj^	22.00 ± 1.13^gi^	29.01 ± 2.82^op^	19.15 ± 0.35^a^	1.43 ± 0.03^bc^	16.06 ± 0.33^gi^
**cic1**	78.4 ± 0.07^f^	42.8 ± 0.21 ^p^	0.5 ± 0.71^op^	16.7 ± 0.00^a^	40.10 ± 0.85^bc^	12.55 ± 0.21^bc^	27.55 ± 0.64^ce^	35.40 ± 1.37^n^	17.87 ± 0.06^b^	1.26 ± 0.08^cd^	14.95 ± 0.3^km^
**cla**	82.6 ± 0.28^a^	48.4 ± 0.35^k^	11 ± 1.41^fh^	13.95 ± 0.07^e^	30.79 ± 0.72^jl^	9.85 ± 0.78^kl^	21.74 ± 0.65^gi^	83.07 ± 1.39^b^	17.68 ± 0.00^bc^	0.59 ± 0.08^ef^	19.45 ± 0.18^b^
**cot1**	77.8 ± 0.00^gh^	48.8 ± 0.28^jk^	46.5 ± 3.54^c^	14.0 ± 0.07^e^	32.09 ± 1.56^hk^	9.85 ± 0.78^ik^	23.05 ± 0.78^fg^	59.53 ± 1.91^e^	16.16 ± 0.07^eh^	0.41 ± 0.01^fg^	14.70 ± 0.09^mn^
**cuc1**	73.6 ± 0.14^op^	47.7 ± 0.21^l^	11.5 ± 2.12^fh^	15.4 ± 0.00^a^	33.55 ± 0.78^gj^	11.10 ± 0.42^eg^	22.45 ± 0.35^gi^	66.31 ± 0.78^d^	11.40 ± 0.14^p^	-0.92 ± 0.02^k^	8.74 ± 0.04^r^
**fsa1**	78.5 ± 0.00^f^	50.6 ± 0.28^h^	2.5 ± 0.71^ln^	12.5 ± 0.07^h^	20.70 ± 0.42^n^	7.00 ± 0.42^n^	13.70 ± 0.00^l^	18.82 ± 1.66^q^	15.14 ± 0.13^km^	1.51 ± 0.06^ab^	17.98 ± 0.20^c^
**gig1**	74.1 ± 0.00^no^	47.5 ± 0.28^lm^	1.0 ± 0.00^no^	16.2 ± 0.00^a^	40.50 ± 0.99^bc^	13.15 ± 0.07^b^	27.35 ± 0.92^ce^	44.43 ± 1.36^k^	16.60 ± 0.16^df^	1.75 ± 0.05^a^	16.68 ± 0.13^ef^
**gio1**	75.5 ± 0.21^lm^	53.5 ± 0.14^e^	1.5 ± 0.71^mo^	14.7 ± 0.07^d^	30.20 ± 0.71 ^l^	9.65 ± 0.21^jk^	20.55 ± 0.49^l^	26.47 ± 1.72^p^	15.86 ± 0.12^gi^	0.61 ± 0.05^df^	17.41 ± 0.13^d^
**giu1**	76.5 ± 0.21^jk^	45.0 ± 0.07^o^	2.5 ± 0.71^ln^	15.5 ± 0.07^a^	35.25 ± 0.64^fh^	10.80 ± 0.28^fh^	24.45 ± 0.35^ef^	34.17 ± 1.19^n^	16.19 ± 0.23^eh^	0.22 ± 0.09^gh^	16.55 ± 0.22^eg^
**ing2**	77.2 ± 0.14^hi^	49.3 ± 0.07^ij^	3.5 ± 0.7^jn^	16.1 ± 0.00^a^	39.45 ± 0.35 ^cd^	12.30 ± 0.14^bd^	27.15 ± 0.21^cd^	43.47 ± 0.51^k^	16.32 ± 0.09^eg^	0.12 ± 0.06	15.65 ± 0.08^ij^
**mar2**	75.4 ± 0.07^lm^	51.5 ± 0.07^g^	14.0 ± 1.4^fg^	12.9 ± 0.00^g^	20.20 ± 1.70 ^n^	7.75 ± 0.35^no^	12.45 ± 1.34^lm^	78.64 ± 1.79^c^	15.77 ± 0.10^hi^	1.39 ± 0.0^bc^	17.08 ± 0.13^de^
**mla1**	75.1 ± 0.14^m^	46.9 ± 0.14^m^	7.0 ± 1.4^hij^	12.9 ± 0.00^g^	18.05 ± 1.061 ^o^	6.75 ± 0.35^m^	11.30 ± 0.71^mn^	91.12 ± 0.52^a^	15.60 ± 0.06^ik^	1.21 ± 0.04^cd^	16.93 ± 0.02^de^
**pao2**	76.9 ± 0.07^ik^	57.4 ± 0.14^b^	66.5 ± 3.54^b^	15.0 ± 0.00^bc^	45.40 ± 0.71^a^	12.50 ± 0.42^bc^	32.90 ± 0.28^a^	50.88 ± 0.77^ij^	14.98 ± 0.06^lm^	1.15 ± 0.01^d^	11.64 ± 0.05^q^
**rea4**	73.7 ± 0.14^no^	41.4 ± 0.42^q^	9.0 ± 1.41^gi^	15.0 ± 0.07^bc^	31.75 ± 0.35^jl^	10.80 ± 0.42^fh^	20.95 ± 0.07^hi^	52.12 ± 0.53^hi^	16.12 ± 0.13^fh^	1.01 ± 0.03^d^	18.03 ± 0.11^c^
**reg1**	76.4 ± 0.21^k^	56.6 ± 0.28^c^	4.0 ± 1.41^jm^	16.3 ± 0.00^a^	39.00 ± 0.28^cd^	11.35 ± 0.07^dg^	27.65 ± 0.21^c^	42.31 ± 0.42^kl^	16.52 ± 0.05^df^	0.96 ± 0.01^d^	15.18 ± 0.03^im^
**rus1**	77.1 ± 0.07^ik^	57.4 ± 0.21^b^	10 ± 1.41^fi^	13.3 ± 0.07^f^	24.90 ± 0.28 ^m^	8.80 ± 0.28^l^	16.10 ± 0.00^k^	57.83 ± 0.48^ef^	15.41 ± 0.01^il^	0.83 ± 0.06^de^	16.26 ± 0.22^fh^
**sam3**	80.2 ± 0.14^d^	51.2 ± 0.2^gh^	6.5 ± 0.71^hk^	14.9 ± 0.07^ce^	38.80 ± 0.57 ^cd^	11.65 ± 0.07^cf^	27.15 ± 0.49^ce^	48.45 ± 0.75^j^	16.50 ± 0.45^df^	0.45 ± 0.03^fg^	16.68 ± 0.20^ef^
**sco4**	73.3 ± 0.21^p^	44.7 ± 0.14^o^	0.0 ± 0.00^p^	16.7 ± 0.07^a^	38.45 ± 0.64^ce^	12.10 ± 0.14^cd^	26.35 ± 0.78^ce^	54.22 ± 0.76^gi^	17.29 ± 0.09^c^	0.82 ± 0.03^de^	18.37 ± 0.06^c^
**sim**	79.1 ± 0.14^e^	52.3 ± 0.28^f^	3.0 ± 1.41^kn^	14.65 ± 0.07^d^	31.63 ± 0.56^jl^	9,30 ± 0.14^jl^	22.33 ± 0.42^gi^	86.30 ± 0.25^b^	17.29 ± 0.18^c^	0.60 ± 0.04^ef^	20.06 ± 0.29^a^
**tim1**	75.3 ± 0.28^lm^	33.5 ± 0.00^s^	9.0 ± 1.41^gi^	13.5 ± 0.14^f^	26.95 ± 0.50 ^m^	8.55 ± 0.07^l^	18.40 ± 0.42^j^	30.60 ± 1.27^o^	19.29 ± 0.11^a^	1.87 ± 0.01^a^	14.81 ± 0.02^lm^
**tre2**	80.3 ± 0.14^cd^	47.5 ± 0.21^lm^	26.5 ± 2.12 ^e^	11.9 ± 0.07^n^	14.00 ± 0.28 ^p^	5.30 ± 0.14^p^	8.70 ± 0.14^o^	67.14 ± 0.66^d^	15.25 ± 0.01^jm^	-0.05 ± 0.02^i^	16.28 ± 0.01^fh^
**trin**	80.3 ± 0.14^cd^	56.5 ± 0.21^c^	7.0 ± 2.83^hj^	14.80 ± 0.00^ij^	32.35 ± 1.34 ^il^	10.70 ± 0.42^gi^	21.65 ± 0.92^gi^	83.60 ± 0.68^b^	16.28 ± 0.29^eg^	0.70 ± 0.16^j^	15.74 ± 0.17^hj^
**tri2**	82.3 ± 0.07^a^	39.3 ± 0.07^r^	96.0 ± 1.41^a^	8.5 ± 0.04^k^	15.00 ± 0.14^p^	5.40 ± 0.14^p^	9.60 ± 0.28^no^	77.33 ± 0.21^c^	11.24 ± 0.04^p^	−0.69 ± 0.28^j^	13.49 ± 0.11^p^
**urr1**	80.7 ± 0.00^c^	45.7 ± 0.14^n^	38.0 ± 1.41^d^	11.5 ± 0.07^j^	18.85 ± 0.07^no^	6.20 ± 0.00^o^	12.65 ± 0.07^lm^	38.99 ± 0.23^lm^	15.75 ± 0.18^hi^	0.24 ± 0.0^gh^	11.19 ± 0.16^q^
**val2gl**	82.3 ± 0.28^a^	59.2 ± 0.21^a^	7.0 ± 1.41^hj^	15.7 ± 0.00^a^	37.15 ± 0.07^df^	11.50 ± 0.14^dg^	25.65 ± 0.07^de^	34.05 ± 0.13^n^	16.84 ± 0.01^d^	0.84 ± 0.0^de^	15.34 ± 0.31^jl^

Data are expressed as the mean value ± standard errors of means; different letters denote significant differences between treatments at the at *p* ≤ 0.001, according to the Tukey’s test.

**Table 2:** Summary statistics of genetic variation obtained by the wheat 90k single nucleotide polymorphisms (SNP) array in 27 landraces and 2 historical varieties (Cappelli and Trinakria), belonging to the Sicilian wheat germplasm. The modern varieties Claudio and Simeto were also added as references, while the hexaploid Cuccitta landrace (*T. aestivum* L.) was included as an outgroup.

Parameters	Values
N ^†^	32
Total number of loci	81,587
No. of failed loci	5,594
No. of monomorphic loci	41,926
No. of used loci	18,170
No. of polymorphic loci	13,528
MAF ^#^	0.232

^†^ Sample size, ^#^ minor allele frequency.

**Table 3:** List of wheat samples characterized by 90K SNP array, agro-morphological, phenological, and quality-related traits.

ID	Accession	Origin
bb2	Bufala bianca	In collection at the ESS* and sampled in ‘99-’04 on farm in Randazzo (Catania)
bd3	Bidì	IPK TRI 26213, in collection at the ESS* since 2004 (selection from Tunisian landrace Bidì, line AP4)
bia1	Biancuccia	In collection at the ESS* and sampled in ‘99-’04 on farm in Salemi (Trapani)
bivc	Casedda Bivona	In collection at the ESS* and sampled in ‘99-’04 on farm in S. Stefano Quisqinia (Agrigento)
bnc2	Bufala nera corta	IPK3517, in collection at the ESS* since 2004
brc-b1	Bufala rossa corta b	In collection at the ESS* and sampled in ‘99-’04 on farm in Randazzo (Catania)
capp1	Cappelli	Historical variety - Old collection of ESS* (selection from Tunisian landrace Jenah Rhetifah)
cas1pu	Castiglione pubescente	CGN 8213, in collection at the ESS* since 2004
cic1	Ciciredda	In collection at the ESS* and sampled in ‘99-’04 on farm in Maletto (Catania)
cot1	Cotrone	USDA 157975, in collection at the ESS* since 2004
cuc1	Cuccitta	In collection at the ESS* and sampled in ‘99-’04 on farm in Fiumedinisi (Messina)
fsa1	Francesa	Old collection of ESS*
gig1	Gigante	CGN 8206, in collection at the ESS* since 2004
gio1	Gioia	IPK 3851, in collection at the ESS* since 2004
giu1	Giustalisa	USDA 278354, in collection at the ESS* since 2004
ing2	Inglesa	IPK 3519, in collection at the ESS* since 2004
mar2	Margherito	In collection at the ESS* and sampled in ‘99-’04 on farm in Chiaramonte Gulfi (Ragusa) (selection from Tunisian landrace Mahmoudi)
mla1	Martinella	USDA 157971, in collection at the ESS* since 2004
pao2	Paola	In collection at the ESS* and sampled in ‘99-’04 on farm in Randazzo (Catania)
rea4	Realforte	IPK TRI 28452, in collection at the ESS* since 2004
reg1	Regina	Old collection of ESS*
rus1	Russello	In collection at the ESS* and sampled in ‘99-’04 on farm in Randazzo (Catania)
sam3	Sammartinara	USDA 157958, in collection at the ESS* since 2004
sco4	Scorsonera	In collection at the ESS* and sampled in ‘99-’04 on farm in Santa Croce Camerina (Ragusa)
tim1	Timilia	In collection at the ESS* and sampled in ‘99-’04 on farm in Maletto (Catania)
tre2	Trentino	USDA 157965, in collection at the ESS* since 2004
tri2	Tripolino	Old collection of ESS*
urr1	Urria	In collection at the ESS* and sampled in ‘99-’04 on farm in Santa Croce Camerina (Ragusa)
val2gl	Vallelunga glabra	USDA 157979, in collection at the ESS since 2004
trin	Trinakria	Historical variety - Old collection of ESS*(B14 x Capeiti 8)
sim	Simeto	Modern variety (Capeiti 8 × Valnova)
cla	Claudio	Modern variety (Cimmyt selection × Durango) × (IS1938 × Grazia)

* Experimental Sicilian Station for Durum Wheat (Caltagirone, Catania)

## Supplementary Materials

The following are available online at https://www.mdpi.com/2223-7747/8/5/116/s1; Table S1: Morphological descriptors utilized for characterizing the Sicilian wheat germplasm collection (UPVO/CPVO, 2011); Table S2. One-way ANOVA for grain and wholemeal flour qualitative parameters of Sicilian wheat germplasm collection; Table S3: Nei genetic distance evaluated among the main clusters obtained by the phylogenetic analysis; Table S4: Average Dissimilarity (AD) values for the first four PCoA vectors; Table S5: Iterative AMOVA results. The changes in genetic differentiation with the increased selection of accessions with the highest Average Dissimilarity (AD) values are shown; Table S6: Iterative AMOVA results. The changes in genetic differentiation with the increased removal of accessions with the lowest Average Dissimilarity (AD) values are highlighted; Table S7: SNP profiles used for analysis; Figure S1: Gluten index in old and modern durum wheat germplasm. Different letters indicate significant differences at *p* < 0.01; Figure S2: (A) Person correlation matrix of 11 quality-related traits. Positive correlations are displayed in blue and negative correlations in red color. Color intensity and the circle sizes are proportional to the correlation coefficients. The significant correlations (*p* < 0.05) are highlighted. (B) Scatter plot matrix with the correlation coefficients between variables and their significance levels. The distribution of each variable is shown on the diagonal; the bivariate scatter plots with a fitted line are displayed on the bottom of the diagonal; the correlation values and the significance level are highlighted as stars, on the top of the diagonal. Asterisks indicate significance levels. Figure S3: Principal Coordinates Analysis (PCoA) developed with the SNP array. Cyan points: 27 landraces belonging to ancient wheat germplasm of Sicily; brown points: 2 historical varieties (Cappelli and Trinakria); red points: 2 modern varieties (Simeto, and Claudio); and blue points: The outgroup (Cuccitta).
