# Supplementary material for: High-Throughput Genotype, Morphology, and Quality Traits Evaluation for the Assessment of Genetic Diversity of Wheat Landraces from Sicily
Source: Plants (Basel). 2019 Apr 30;8(5):116. doi: 10.3390/plants8050116 (PMC6572038; doi:10.3390/plants8050116)
Supplement: Supplementary file 1 [file plants-08-00116-s001.zip › supplementary files/Figure S1.docx]

**
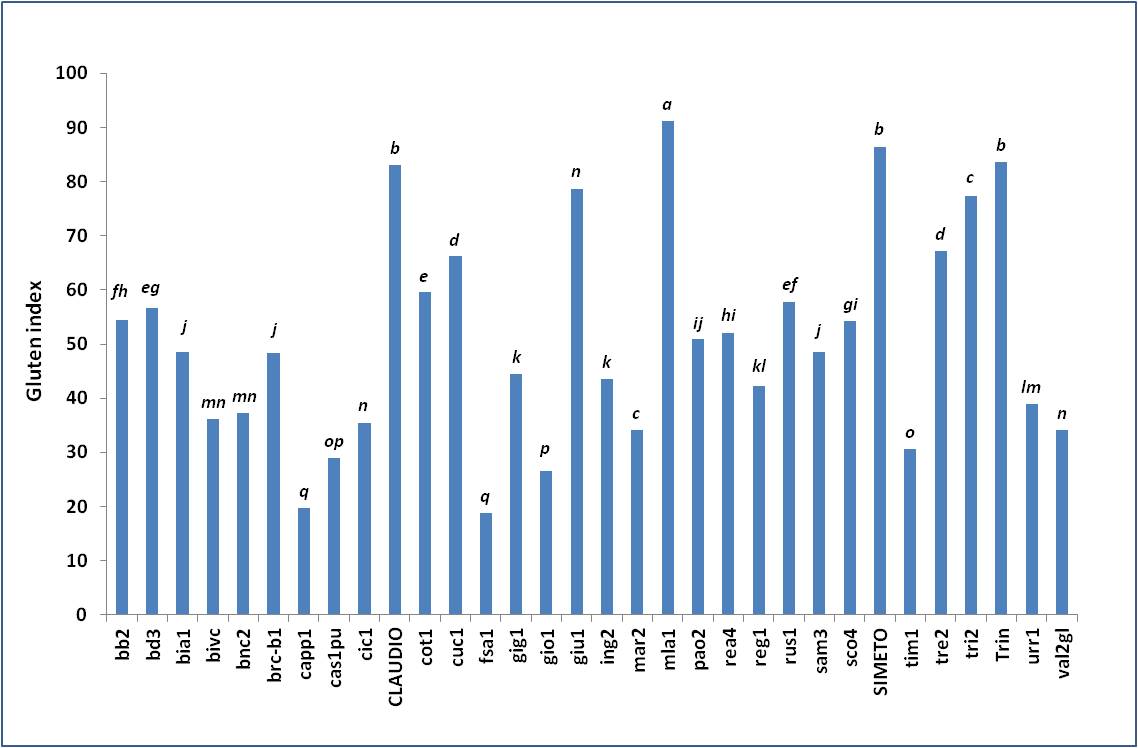
**

**Figure S1.** Gluten index in old and modern durum wheat germplasm. Different letters indicate significant differences at *p* < 0.01.
