# Supplementary material for: High-Throughput Genotype, Morphology, and Quality Traits Evaluation for the Assessment of Genetic Diversity of Wheat Landraces from Sicily
Source: Plants (Basel). 2019 Apr 30;8(5):116. doi: 10.3390/plants8050116 (PMC6572038; doi:10.3390/plants8050116)
Supplement: Supplementary file 1 [file plants-08-00116-s001.zip › supplementary files/Figure S2.docx]

**
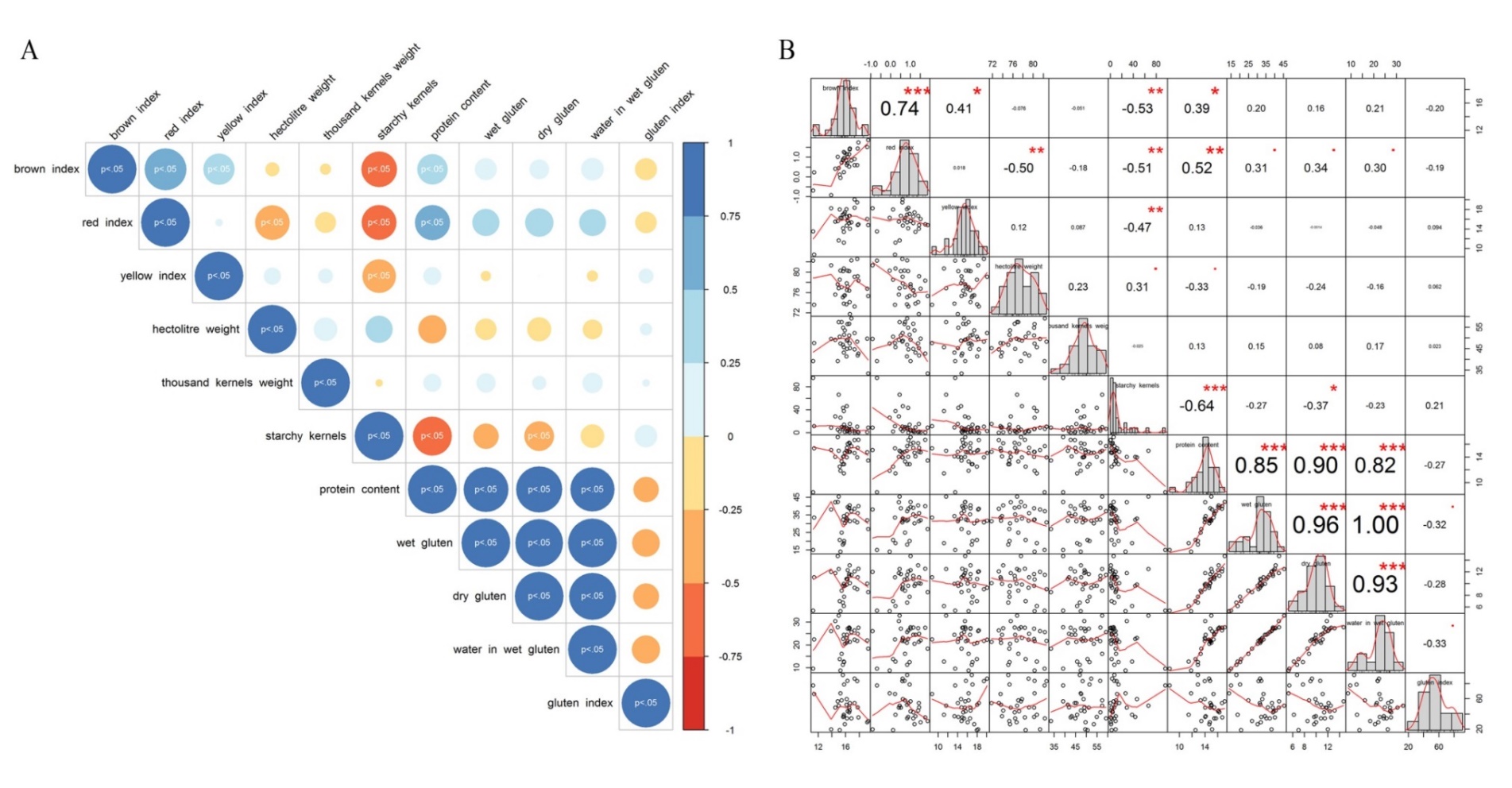
**

**Figure S2.** (**A**) Pearson correlation matrix of 11 qualitative traits. Positive correlations are displayed in blue and negative correlations in red color. Color intensity and the circle sizes are proportional to the correlation coefficients. The significant correlations (*p* < 0.05) are highlighted. (**B**) Scatter plot matrix with the correlation coefficients between variables and their significance levels. The distribution of each variable is shown on the diagonal; the bivariate scatter plots with a fitted line are displayed on the bottom of the diagonal; the correlation values and the significance level are highlighted as stars on the top of the diagonal. Asterisks indicate significance levels.
