# Supplementary material for: High-Throughput Genotype, Morphology, and Quality Traits Evaluation for the Assessment of Genetic Diversity of Wheat Landraces from Sicily
Source: Plants (Basel). 2019 Apr 30;8(5):116. doi: 10.3390/plants8050116 (PMC6572038; doi:10.3390/plants8050116)
Supplement: Supplementary file 1 [file plants-08-00116-s001.zip › supplementary files/Figure S3.docx]

**
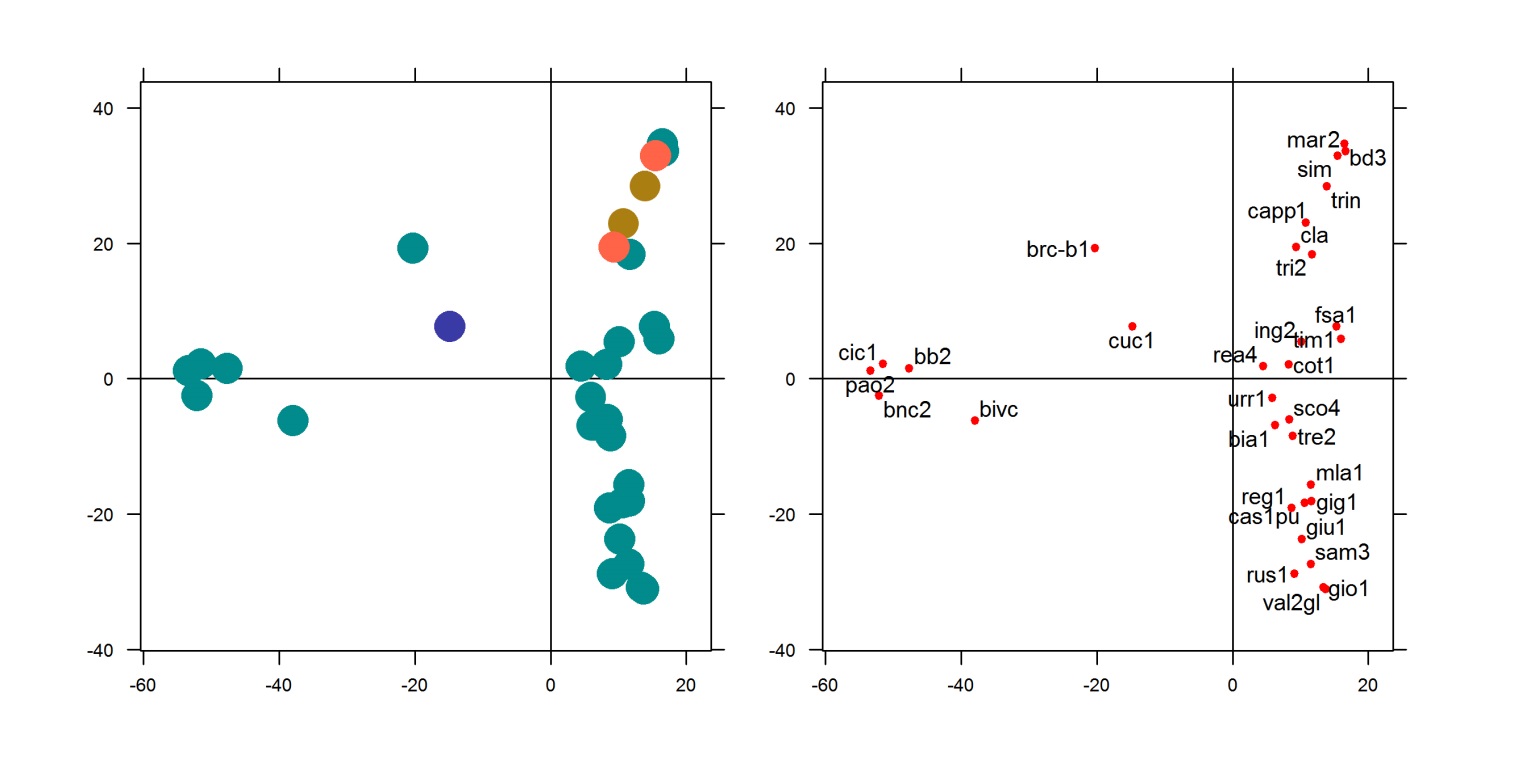
**

**Figure S3.** Principal Coordinates Analysis (PCoA) developed with the SNP array. Cyan points: 27 landraces belonging to ancient wheat germplasm of Sicily; brown points: 2 historical varieties (Cappelli and Trinakria); red points: 2 modern varieties (Simeto, and Claudio); and blue points: The outgroup (Cuccitta).
