# Supplementary material for: High-Throughput Genotype, Morphology, and Quality Traits Evaluation for the Assessment of Genetic Diversity of Wheat Landraces from Sicily
Source: Plants (Basel). 2019 Apr 30;8(5):116. doi: 10.3390/plants8050116 (PMC6572038; doi:10.3390/plants8050116)
Supplement: Supplementary file 1 [file plants-08-00116-s001.zip › supplementary files/Table S3.docx]

**Table S3.** Nei genetic distance evaluated among the main clusters obtained by the phylogenetic analysis

|  | **Cluster A** | **Cluster B** | **Cluster C** | **Cluster D** | **Outgroup** |
| --- | --- | --- | --- | --- | --- |
| **Cluster A** | - |  |  |  |  |
| **Cluster B** | 0.1552 | - |  |  |  |
| **Cluster C** | 0.2154 | 0.1857 | - |  |  |
| **Cluster D** | 0.3581 | 0.2945 | 0.4585 | - |  |
| **Outgroup** | 0.4663 | 0.4320 | 0.4965 | 0.4560 | - |
